# TransOdara study: the challenge of integrating methods, settings and procedures during the COVID-19 pandemic in Brazil

**DOI:** 10.1590/1980-549720240002.supl.1

**Published:** 2024-08-19

**Authors:** Maria Amelia de Sousa Mascena Veras, Thiago Felix Pinheiro, Lenice Galan, Laio Magno, Andréa Fachel Leal, Daniela Riva Knauth, Ana Rita Coimbra Motta-Castro, Rita Suely Bacuri de Queiroz, Philippe Mayaud, Daniel Jason McCartney, Gwenda Hughes, Camila Mattos dos Santos, Leonardo Bastos, Katia Cristina Bassichetto, Sandro Sperandei, Claudia Renata dos Santos Barros, Rodrigo Calado da Silva, Francisco Inácio Bastos, Maria Inês Costa Dourado

**Affiliations:** I Santa Casa de São Paulo, School of Medical Sciences – São Paulo (SP), Brazil.; II Núcleo de Pesquisa e Direitos Humanos em Saúde da População LGBT+ (NUDHES) – São Paulo (SP), Brazil.; III Universidade do Estado da Bahia, Department of Life Sciences – Salvador (BA), Brazil.; IV Universidade Federal do Rio Grande do Sul – Porto Alegre (RS), Brazil.; V Universidade Federal de Mato Grosso do Sul – Campo Grande (MS), Brazil.; VI Fundação Oswaldo Cruz, Instituto Leônidas e Maria Deane – Manaus (AM), Brazil.; VI London School of Hygiene & Tropical Medicine, Faculty of Infectious & Tropical Diseases, Department of Clinical Research – London, United Kingdom.; VIII London School of Hygiene & Tropical Medicine, Faculty of Epidemiology and Public Health, Department of Infectious Diseases – London, United Kingdom.; IX Fundação Oswaldo Cruz, Scientific Computing Program – Rio de Janeiro (RJ), Brazil.; X Western Sydney University, Translational Health Research Institute – Penrith, Australia.; XI Universidade de São Paulo, Instituto Butantã – São Paulo (SP), Brazil.; XII Santa Casa Instituto de Pesquisa – São Paulo (SP), Brazil.; XIII Fundação Oswaldo Cruz, Institute of Scientific and Technological Communication and Information in Health – Rio de Janeiro (RJ), Brazil.; XIV Universidade Federal da Bahia, Institute of Public Health – Salvador (BA), Brazil.

**Keywords:** Transgender persons, Syphilis, HIV, Sexually transmitted infections, Sampling studies, Respondent-driven sampling, Implementation science

## Abstract

**Objective:**

Sexually transmitted infections (STIs) disproportionately affect transgender women and *travestis* (TGW), who often lack access to healthcare due to stigma and discrimination. We describe the approach and methodology of a study investigating the prevalence of syphilis, HIV, hepatitis A, B, and C, *Neisseria gonorrhoeae* (NG), *Chlamydia trachomatis* (CT), and human papillomavirus (HPV) among TGW, as well as their knowledge and perceptions regarding syphilis, to better inform policies to curb STIs among this vulnerable population.

**Methods:**

TransOdara was a multicentric, cross-sectional study conducted among TGW in five capital cities from major Brazilian regions between December 2019 and July 2021. Self-identified transgender women and *travestis* aged >18 years were recruited using respondent-driven sampling after a qualitative formative phase, completed an interviewer-led questionnaire, were offered a physical examination, and were also asked to provide samples from multiple sites to detect various STIs, starting vaccination and treatment when indicated.

**Results:**

A total of 1,317 participants were recruited from the five study locations: Campo Grande (n=181, 13.7%), Manaus (n=340, 25.8%), Porto Alegre (n=192, 14.6%), Salvador (n=201, 15.3%), and São Paulo (n=403, 30.6%). The recruitment period varied at each study location due to logistic constraints imposed by the COVID-19 pandemic.

**Conclusion:**

Despite the enormous challenges posed by the co-occurrence of the COVID-19 pandemic and field work targeting a vulnerable, elusive, and scattered population, the TransOdara project has been effectively implemented. Caveats did not preclude 1,300 TGW from being interviewed and tested, amid a significant epidemic that disrupted health services and research projects in Brazil and worldwide.

## INTRODUCTION

Sexually transmitted infections (STIs) continue to be a major public health concern worldwide, impacting quality of life and causing serious morbidity and mortality. According to the World Health Organization (WHO), over a million curable STIs are acquired every day, primarily caused by *Chlamydia trachomatis* (CT), *Neisseria gonorrhoeae* (NG), *Treponema pallidum* (syphilis), and *Trichomonas vaginalis*, while an unacceptably high number of people still die from HIV and hepatitis B or C^1^.

Recently, strategies have been proposed to reduce the global burden of STIs, including developing rapid point-of-care tests, which provide results soon after sample collection to avoid missing the opportunity for prompt treatment and care^2^. Such strategies are especially relevant to reduce incidence among populations who face barriers to access care and treatment in health facilities.

Despite these efforts, the prevalence of STIs has not decreased, and there have been recent outbreaks of new infections transmitted through sexual contacts, such as Mpox, *Shigella sonnei*, *Neisseria meningitidis*, Ebola, and Zika, making it challenging to control these diseases and their consequences^1^. Furthermore, antimicrobial resistance poses a significant global public health threat, particularly for NG, as it could lead to even more untreatable infections^3-5^.

In Brazil, data on the prevalence of the most common STIs are limited, likely due to the syndromic management of STIs implemented by the public health system since the early 1990s, resulting in limited etiological STI reporting/surveillance to diagnose STIs^6^.

Transgender women and *travestis* (TGW), used here as an umbrella term that includes all individuals who self-identify with a gender identity different than the male sex assigned at birth, are at a higher risk of HIV and other STIs. However, limited data are available on STIs other than HIV for this population, especially in lower- and middle-income countries, such as Brazil^7,8^.

The available data suggest STIs rates in TGW in Brazil are likely to be high^10,11^. The determinants of STI acquisition and transmission overlap with those for HIV, including condomless receptive anal intercourse^12^. Other important risk factors for HIV infection in TGW are psychological and physical abuse resulting from gender non-conformity^13^, young age, black race/skin color, and history of sex work and/or cocaine use. However, more robust, and timely data are needed on the frequency and risk factors of non-HIV STIs in TGW.

Stigma and discrimination are significant obstacles to accessing healthcare services, leading to poorer health outcomes for TGW^9,10^, who frequently report negative experiences in healthcare settings, such as insensitive language or refusal of care, and healthcare providers often feel unprepared to provide appropriate care^11^. This lack of proper gender-affirming care likely inhibits transgender people from seeking testing, diagnosis, and treatment for STIs, as well as other necessary healthcare.

There is a need for robust data on the epidemiology of STIs in TGW and an understanding of the health disparities they experience to inform development of effective prevention and treatment services and strategies^12^.

To address these gaps, we established the TransOdara study with the following aims:

1. To estimate the prevalence of STIs, including syphilis, HIV, NG, CT, human papillomavirus (HPV), hepatitis A virus (HAV), hepatitis B (HBV), and hepatitis C (HCV), among transgender women and *travestis* in each of the five macro-regions of Brazil;
2. To demonstrate the feasibility of implementing point-of-care treatment and prevention in the Brazilian National Health System (in Portuguese, *Sistema Único de Saúde* — SUS); and
3. To understand meanings attributed to syphilis and experiences in health care services among TGW.

Here, we present the TransOdara study methodology and procedures. We describe some of the challenges faced while establishing the study during the COVID-19 pandemic and how these were addressed.

## METHODS

TransOdara was a multi-centric, cross-sectional study conducted in five Brazilian capital cities: Campo Grande, Manaus, Porto Alegre, Salvador, and São Paulo between December 2019 and July 2021, among transgender women and *travestis*.

This mixed-methods study had a convergent design with a simultaneous bidirectional framework for data merging analytics, which involved an interactive consideration of both qualitative and quantitative perspectives^13^.

### Qualitative research

**Figure 1:**
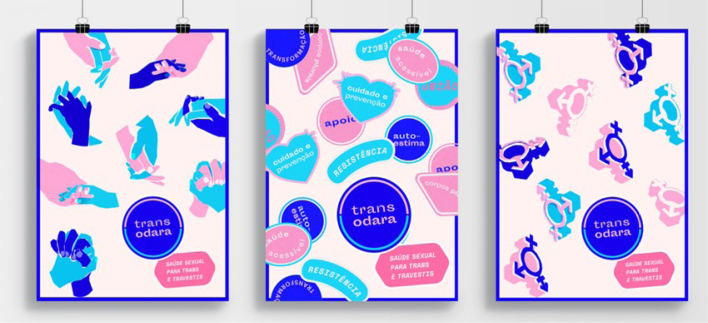
Visual production – art produced for communication, according to the imagery project of the research.

The study began with a formative phase using complementary research methods and techniques. First, social venues and institutions with services for transgender people were mapped and visited to obtain information about the social networks of TGW and their acquaintances to inform the selection of ‘seeds’ for the quantitative component in each study site. Then we identified potential participants, indicated by key-informants, and conducted focus groups to: understand the meanings attributed by transgender women and *travestis* to syphilis and other STIs; identify their knowledge regarding prevention, testing, and treatment of these infections; evaluate the acceptability of different testing and treatment strategies; and understand their experience with health services. In São Paulo, a focus group was initially convened with TGW to name and develop the study’s visual identity from their semantic and aesthetic references, and according to their expectations for a health project (Figure 1).

Another qualitative component was carried out with participants already included in the quantitative component (except in Salvador, where both qualitative stages were carried out simultaneously). During this stage, in-depth interviews were undertaken with participants with different levels of schooling, both with those who reported a previous syphilis diagnosis and those who did not. The interviews aimed to understand the vulnerability of transgender women and *travestis* to STIs, the meanings attributed to such infections, their access (or lack of) to health facilities, and their itineraries within the health care system, with a focus on identifying barriers to the treatment of STIs.

### Quantitative research

#### Sampling procedures and sample size

A sample size of 1,280 transgender women and *travestis* was calculated *a priori* to provide an estimate of syphilis prevalence with standard errors for each site using an appropriate method where the population size is unknown^14^. The sample size for each site was calculated to estimate the prevalence of active syphilis, considering titers >1:8 on the VDRL and the size of the respective cities, as shown in Table 1.

**Table 1:** Sample size for each site and respective standard error.

Site	Sample size	Standard error (%)
Manaus	300	3.7
Salvador	200	4.7
Campo Grande	180	5.0
Porto Alegre	200	4.8
São Paulo	400	3.1

Operational issues and budgetary constraints were important considerations in the choice of active syphilis as the primary outcome for sample size calculations. Previous studies suggested other STIs might be less prevalent than syphilis, leading to much larger sample sizes. Clearly, our choice might result in less than optimal precision of prevalence estimates for rare infections^15^. The impact of this limitation on estimates of specific STIs will be discussed in the relevant analytical papers.

#### Recruitment

Respondent-driven sampling (RDS) was used to recruit TGW across the five study sites. RDS is an appropriate approach for recruiting this underserved population^16^ and has been used for the recruitment of TGW in other studies in Brazil^17^.

During formative research in each study site, potential ‘seeds’ (seven to nine) were selected through in-depth interviews and focus groups with members of the transgender women and *travestis* community, who were initially contacted by outreach workers/peers already working with the local research teams. The criteria for choosing ‘seeds’ included having links to a large social network of potential participants and ensured sufficient diversity across characteristics of interest such as education level and engagement in sex work.

Each seed received a limited number of coupons (5–6) to distribute to others in their social network, and subsequent participants received a similar number of coupons each to distribute. RDS methodology requires that participants refer people they know and with whom they maintain a social relationship. Participants were instructed to invite others who self-identified as part of the same population of interest as the study. The number of coupons distributed to each participant, six, was established to maximize study recruitment and was based on previous research experience with TGW. Recruitment chains were tracked using a “coupon manager”, with each participant given a unique number that identified the research site, individual identification number, and recruitment string.

Eligible participants were 18 years or older, assigned male sex at birth, self-identified with feminine gender identity (including *travesti*, woman, transgender woman, agender, or other female identification), resided in the metropolitan area of the study sites, and presented a valid study coupon.

Participants were reimbursed for food and transportation expenses and were required to provide informed consent to join the study.

Each of the study sites could adopt recruitment strategies most appropriate to their context without compromising the study’s objectives and general design. Contrary to the other sites, in São Paulo, the TransOdara study was nested within the TransNacional cohort study^18^, which also used the RDS methodology for recruitment. Cohort participants were invited to participate in the TransOdara study and were scheduled for the procedures proposed by the new study.

Although the pandemic impeded all the planned on-site training and supervision, in all study sites researchers had previous experience conducting other RDS studies, facilitating online training and supervision of all research activities.

#### Data collection

All participants completed specific data collection forms covering:

- eligibility;
- acceptability of sample collection; and
- a standard interviewer-led questionnaire for sociodemographic information with over two hundred questions related to gender-affirming procedures, stigma and discrimination, incarceration, mental health, alcohol and substance use, sexual behavior, access to healthcare and HIV/STI tests, pre-exposure prophylaxis (PrEP) and post-exposure prophylaxis (PEP) for HIV prevention, and STI symptoms experienced in the past six months and at the time of the study visit.

Face-to-face interviews were done by well-trained staff using laptops.

#### Clinical and laboratory procedures

The participants were asked to provide biological samples from multiple body sites for STI screening voluntarily, with the right to refuse any of the tests. Blood samples were collected and tested for HIV, syphilis, and hepatitis (A, B, C) infections, using current guidelines of the Brazilian Ministry of Health and with products registered with and approved by the National Sanitary Surveillance Agency (in Portuguese, *Agência Nacional de Vigilância Sanitária* — ANVISA). Rapid tests for detection of anti-HIV-1/2, anti-HCV, anti-HBsAg, and anti-*Treponema pallidum* were conducted during the study visit, followed by laboratory testing for confirmation and to ascertain recency of infection.

Urine, anorectal, and oropharyngeal samples were tested for CT and NG using Abbott RealTime CT/NG assay (Des Plaines, IL, USA), which has previously demonstrated high accuracy for each of those anatomical sites for each pathogen^19^. The Seegene Anyplex II HPV28 Detection assay (Seoul, Republic of Korea) was used to detect HPV DNA and identify specific genotypes present in swabs of the perianal region and the external genitals, with accuracy mostly demonstrated for cervical samples in cisgender females, but deemed acceptable for detection of HPV genotypes in the anal canal^20^. Participants could choose whether these samples were self-collected or provider-collected, with instructional diagrams specifically developed for the study, taking into consideration transgender bodies, to guide participants with self-collection. Detailed procedures, and acceptability of self or provider sample collection were published elsewhere^21^.

Each participant was asked for permission to undergo a physical examination by a study clinician to observe and report signs of infection using a standardized case report form, irrespective of any reported symptoms, and could opt out of all or any examinations. This included a general examination of the skin, oropharynx, and axillary and groin lymph nodes (to detect possible signs of syphilis, warts, ulcers, inflammation, and adenomegaly); a genital examination (to detect the presence of genital discharge, warts, and ulcers); and an anal examination (to detect the presence of anal discharge, warts, and ulcers). The genital examination was based on the genitalia present (penis and scrotum or a neovagina following surgery).

Overall, both clinical and laboratory exams were well-accepted by the interviewees and the number of refusals was negligible (i.e. 98.5% consented to have biological samples collected).

Where necessary, participants were referred for treatment of any condition evidenced by laboratory tests or clinical examinations. Vaccination status was assessed, and participants were referred to receive vaccines at SUS services as indicated. Participants in all cities followed the same steps regarding being offered tests, exams, and vaccines. Additionally, standard operational procedures (SOPs) were developed to ensure homogeneity across the five study sites. A data manager worked throughout the study period to check on the quality of collected data and request corrections or completeness whenever necessary.

#### Data storage and cleaning

All data, including responses from interviewer-led questionnaires and case report forms, were collected and managed as a single-entry using REDCap^22^, a secure, web-based platform designed to support validated data collected for research studies, hosted at the Faculty of Medical Sciences of Santa Casa de São Paulo. Data cleaning and statistical analysis were performed using R^23^. To estimate the prevalence of STIs of interest, each city was treated as a cluster due to peer-chain recruitment and the diversity of networks. The RDS weights were not used in the analysis, as recent studies have shown they do not improve the model performance and could introduce further uncertainty, depending on the underlying network structure^24,25^.

#### Ethical aspects

The protocol for this study was approved by the Research Ethics Committee (CEP) of the Santa Casa de São Paulo (Certificate of Presentation for Ethical Appreciation — CAAE 05585518.7.0000.5479), and subsequently by the CEPs of the other participating institutions: Instituto Leônidas and Maria Deane — ILMD/Fiocruz — Manaus; STD/AIDS Reference and Training Center, Instituto Adolfo Lutz; Municipal Secretary of Health of Porto Alegre; Federal University of Bahia; Federal University of Mato Grosso do Sul — UFMS, Federal University of Health Sciences of Porto Alegre — UFCSPA, and Federal University of Rio Grande do Sul — UFRGS.

Written informed consent was obtained from all individual participants included in the study. Individuals who identified with a transgender-feminine identity were involved in the design and implementation of the study and led the recruitment of participants using RDS.

## RESULTS

A total of 1,317 participants were recruited from the five study locations: Campo Grande (n=180, 13.7%), Manaus (n=333, 25.8%), Porto Alegre (n=191, 14.6%), Salvador (n=207, 15.3%), and São Paulo (n=406, 30.6%). The recruitment period varied at each study site, due to different lengths of time to get ethical approval and to the consequences of the epidemiological waves of the COVID-19 pandemic. In different phases of the epidemic curve, measures such as advising people to stay at home, restricting access to primary health care facilities (where four out of five sites were) to emergencies, and closing down university campuses, among other measures, directly affected the research activities.

The age of participants ranged from 18 to 68 years, with a mean age of 30 years (standard deviation — SD=9.9), varying by cities: 29 — SD=10.7 (Campo Grande), 30 — SD 9.3 (Manaus), 32 — SD=9.9 (Porto Alegre), 28 — SD=9.6 (Salvador) e 33 — SD=9.7 (São Paulo). A median age of 31 years (interquartile range — IQR 24–38). Most participants identified as having a ‘mixed’ race/skin color (44%), with similar proportions reporting ‘black’ or ‘white’ (27 and 26%, respectively). The majority of participants identified as transsexual woman (56.3%) or *travesti* (29.8%).

Less than one-third (29.3%) reported changing their name on any official document. Over one-third of participants reported no religion (36%). Catholicism was the most reported religion (26%), followed by Afro-Brazilian ones (22%). The majority reported a secondary-level education (58%), with one-quarter (25%) reporting only primary-level or no education.

A substantial percentage of participants (37.6%) were living in unstable conditions (i.e., shelters, sleeping in their workplace) including homelessness (1.8%). Employment status varied, the largest proportion being unemployed (22.3%), followed by being a sex worker (21.3%) or self-employed (16.1%). Only a small proportion reported being formally employed (8.4%). The monthly income of most participants (57%) was up to one minimum wage — in Brazilian *reais* (BRL), approximately 1,000.00 (R$ 998.00 in 2019, 1,100.00 in 2021, or approximately US$ 200).

Over one-quarter (27%) reported undergoing some transition-related procedure or surgery, while a very small proportion (1.7%) reported having a neovagina after undergoing surgery to remove their penis and scrotum. Most participants (86%) have used hormones before, while a little less than half (48%) were using gender-affirming hormones.

The study found high levels of discrimination and violence, with most participants reporting that they had experienced some kind of discrimination (85.0%) or forced sex (51%). Almost one-quarter (23%) reported having being arrested, and among those less than 1% (0.8%) were placed in a cell dedicated to LGBTQI+ persons.

Most participants reported their sexual orientation as heterosexual (78.7%), and their partnership status as single (69.9%). In the past six months, less than half reported having a steady sex partner (48.6%) or casual sex partners (44.0%). While two-fifths (39.7%) indicated having at least one commercial sex partner in the past six months, one-fifth (21.3%) reported sex work as their main source of income. Most participants reported having engaged in transactional sex (73.7%).

The participants’ characteristics by site and the total sample are presented in Table 2.

**Table 2:** Sociodemographic characteristics of transgender women and *travestis* participating in the TransOdara study, according to cities of study. December 2019–July 2021.

Characteristics/sites	Research sites and Brazil					
	Campo Grande (n=180)	Manaus (n=333)	Porto Alegre (n=191)	Salvador (n=207)	São Paulo (n=406)	Total (n=1,317)
	n (%)	n (%)	n (%)	n (%)	n (%)	n (%)
Sociodemographic data						
Gender identity						
Woman	12 (6.8)	4 (1.2)	21 (11)	22 (11)	39 (9.6)	98 (7.4)
Transsexual woman	78 (43)	172 (52)	96 (50)	130 (63)	265 (65)	741 (56)
*Travesti*	59 (32.8)	150 (45)	48 (25)	40 (19)	96 (24)	393 (30)
Transsexual	9 (5.0)	0 (0)	18 (9.4)	7 (3.4)	4 (1.0)	38 (2.9)
Other	22 (12.4)	7 (2.1)	8 (4.2)	8 (3.9)	2 (0.5)	47 (3.7)
Tried to change name						
I didn’t feel the need	33 (24)	208 (68)	20 (19)	46 (30)	46 (20)	353 (38)
I didn’t know I had a right	1 (0.7)	30 (9.8)	3 (2.8)	6 (3.9)	4 (1.7)	44 (4.7)
I didn’t know how to do it	6 (4.4)	29 (9.5)	16 (15)	13 (8.6)	13 (5.7)	77 (8.3)
I thought it was too expensive	12 (8.8)	8 (2.6)	9 (8.3)	6 (3.9)	41 (18)	76 (8.2)
I thought it was too complicated	11 (8.1)	11 (3.6)	13 (12)	35 (23)	50 (22)	120 (13)
Other reasons/did not answer	73 (54)	19 (6.2)	47 (44)	46 (30)	76 (33)	261 (28)
Race/skin colour						
White	55 (32)	60 (18)	91 (48)	22 (11)	108 (27)	336 (26)
Black	38 (22)	52 (16)	60 (31)	107 (52)	92 (23)	349 (27)
Yellow	2 (1.2)	12 (3.6)	3 (1.6)	4 (1.9)	5 (1.2)	26 (2.0)
Brown (*parda*)	1 (0.6)	8 (2.4)	1 (0.5)	2 (1.0)	7 (1.7)	19 (1.5)
Indigenous	76 (44)	199 (60)	36 (19)	72 (35)	192 (47)	575 (44)
Religion						
No religion	70 (40)	100 (30)	62 (33)	92 (44)	151 (37)	475 (36)
Afro-Brazilian	24 (14)	37 (11)	83 (44)	73 (35)	69 (17)	286 (22)
Evangelical	18 (10)	33 (9.9)	7 (3.7)	6 (2.9)	52 (13)	116 (8.8)
Catholic	49 (28)	149 (45)	26 (14)	31 (15)	91 (22)	346 (26)
Spiritist	14 (8.0)	12 (3.6)	10 (5.3)	4 (1.9)	35 (8.6)	75 (5.7)
Jewish	0 (0)	0 (0)	0 (0)	0 (0)	2 (0.5)	2 (0.2)
Eastern	1 (0.6)	1 (0.3)	2 (1.1)	1 (0.5)	4 (1.0)	9 (0.7)
Highest educational level						
Up to elementary school	48 (27)	76 (23)	52 (27)	55 (27)	97 (24)	328 (25)
Up to high school and technical course	95 (53)	201 (61)	97 (51)	117 (57)	253 (62)	763 (58)
Higher education and Postgraduate studies	35 (20)	55 (17)	42 (22)	34 (16)	56 (14)	222 (17)
Living conditions						
Lives in own house/apartment	44 (25)	50 (15)	63 (33)	71 (34)	115 (28)	343 (26)
Lives in a rented house or apartment	60 (34)	82 (25)	65 (34)	94 (45)	179 (44)	480 (36)
Lives provisionally with family/friend/on the street	61 (34)	171 (51)	52 (27)	31 (15)	52 (13)	367 (28)
Sexual orientation						
Heterosexual	106 (59)	288 (87)	127 (66)	168 (81)	347 (85)	1,036 (79)
Homosexual, gay, or lesbian	34 (19)	23 (6.9)	17 (8.9)	7 (3.4)	16 (3.9)	97 (7.4)
Bisexual	20 (11)	15 (4.5)	17 (8.9)	10 (4.8)	23 (5.7)	85 (6.5)
Pansexual	15 (8.4)	5 (1.5)	26 (14)	19 (9.2)	16 (3.9)	81 (6.2)
Asexual	1 (0.6)	0 (0)	1 (0.5)	0 (0)	1 (0.2)	3 (0.2)
Main occupation						
Not currently working/unemployed	27 (15)	113 (34)	49 (26)	36 (17)	69 (17)	294 (22)
Employee with a work permit	9 (5.0)	15 (4.5)	16 (8.4)	10 (4.8)	61 (15)	111 (8.4)
Employee without a work permit	23 (13)	56 (17)	10 (5.2)	21 (10)	40 (9.9)	150 (11)
Self-employed	19 (11)	39 (12)	45 (24)	55 (27)	54 (13)	212 (16)
Sporadic jobs/odd jobs	5 (2.8)	37 (11)	7 (3.7)	22 (11)	39 (9.6)	110 (8.4)
Sex worker	54 (30)	42 (13)	38 (20)	57 (28)	89 (22)	280 (21)
Other	42 (23)	30 (9.0)	26 (14)	6 (2.9)	54 (13)	158 (12)
Monthly income						
Mean	1939.8	1228	1264.6	1564.8	1569.8	1499.3
Standard deviation	1840.3	2772.8	1306.1	1480.5	3943.1	2810.8
Income in Brazilian minimum wages						
Up to 1	61 (36)	183 (69)	97 (53)	125 (66)	216 (55)	682 (57)
1–2	66 (39)	61 (23)	50 (27)	41 (22)	129 (33)	347 (29)
2–3	18 (11)	9 (3.4)	21 (12)	11 (5.8)	31 (7.9)	90 (7.5)
4+	24 (14)	11 (4.2)	14 (7.7)	12 (6.3)	17 (4.3)	78 (6.5)
Body change						
Used hormones						
No	37 (21)	78 (23)	31 (16)	17 (8.2)	20 (4.9)	183 (14)
Yes	142 (79)	253 (76)	160 (84)	190 (92)	386 (95)	1,131 (86)
Current use of hormones						
No	87 (61)	159 (63)	82 (51)	77 (41)	186 (48)	591 (52)
Yes	55 (39)	93 (37)	77 (48)	113 (59)	198 (52)	536 (48)
Has injected industrial silicone						
No	117 (65)	293 (89)	141 (74)	152 (73)	248 (61)	951 (72)
Yes	62 (35)	35 (11)	50 (26)	53 (26)	158 (39)	358 (27)
Had some surgery for body change						
No	139 (78)	315 (95)	97 (51)	165 (80)	234 (58)	950 (72)
Yes	40 (22)	14 (4.2)	93 (49)	40 (19)	172 (42)	359 (27)
Had genital surgery						
No	179 (100)	327 (98)	186 (97)	205 (99)	393 (97)	1,290 (98)
Yes	0 (0)	2 (0.6)	5 (2.6)	2 (1.0)	13 (3.2)	22 (1.7)
Was discriminated against						
No	33 (18)	50 (15)	23 (12)	36 (17)	48 (12)	190 (14)
Yes	146 (82)	281 (84)	168 (88)	171 (83)	354 (88)	1,120 (85)
Suffered some aggression						
No	91 (51)	197 (60)	82 (43)	110 (53)	202 (50)	682 (52)
Yes	87 (49)	128 (39)	109 (57)	96 (46)	202 (50)	622 (48)
Was forced to have sex						
No	97 (54)	175 (53)	87 (46)	90 (43)	193 (48)	642 (49)
Yes	82 (46)	153 (47)	103 (54)	116 (56)	213 (52)	667 (51)
Sexual behavior						
Had a stable partner in the past 6 months						
No	90 (51)	229 (69)	83 (43)	86 (42)	184 (45)	672 (51)
Yes	88 (49)	102 (31)	108 (57)	120 (58)	222 (55)	640 (49)
Had a casual partner in the past 6 months						
No	93 (52)	225 (68)	76 (40)	101 (49)	233 (58)	728 (55.6)
Yes	86 (48)	104 (31)	114 (60)	106 (51)	169 (42)	579 (44)
Had a commercial partner in the past 6 months						
No	89 (50)	255 (77)	88 (46)	107 (52)	246 (61)	785 (60)
Yes	90 (50)	75 (23)	101 (53)	99 (48)	158 (39)	523 (40)

## DISCUSSION

The TransOdara study was the first large study in Brazil designed for transgender women and *travestis* testing for eight different sexually-transmitted infections, including HIV, syphilis, hepatitis A, hepatitis B, hepatitis C, HPV, gonorrhea, and chlamydia. Participants characteristics confirm that a substantial number of TGW face social vulnerability, experience discrimination and barriers to access their rights.

The study found that self-sampling for etiological diagnosis of STIs from potential infection sites was highly acceptable among transgender women and *travestis*, and this could be an important tool in the prevention and care of those infections among this population. The acceptability was facilitated by a friendly and well-trained team of providers^21^.

Despite the interruptions and delays caused by the COVID-19 pandemic, the study was successfully carried out. In São Paulo, the only site that had already started (December 3^rd^ of 2019) when the pandemic emerged in Brazil, the study reached half of the target sample when the fieldwork was interrupted on March 18, 2020. The study was restarted four months later, but it was not possible to maintain the original chain of RDS recruitment, and there were limitations on the use of network analysis. The study was resumed under a rigid protocol, including prior contact by phone to assess suspected SARS-CoV-2 infection in participants. Data collection lasted up to October 29^th^ of 2020.

Although RDS can efficiently recruit large numbers of participants in a relatively short period and at a low cost, it can also result in sample and selection bias. As the recruitment process relies on social networks, the sample may need to be more representative and could exclude specific subgroups. Participants who chose to take part may also have specific characteristics that differ from those who did not. In this study, for instance, depending on the ‘seeds’ selected for recruitment at each site, those engaged in sex work may be overrepresented in some or all locations. Additionally, differences in population characteristics across multiple sites can make it challenging to compare results, and careful interpretation is necessary, especially when reporting overall prevalence. Mitigating these biases can be achieved through various statistical approaches to account for the effects of RDS^24^.

To our knowledge, this is the first large multicenter study targeting a vulnerable and scattered transgender population amid the COVID-19 pandemic. We are candid about the disruptions imposed by the pandemic and the biases that fractured referral chains may cause. As discussed by Christenfeld et al.^26^, science should not be viewed as an inexhaustible source of “magic tricks”. However, fractured referral chains will never be pristine networks, and a deadly epidemic must be addressed primarily, as was the case of this study.

The participants gave significant positive feedback about services received from the point-of-care strategy that integrates testing, treatment, and prevention, including PrEP initiation and vaccines (data not shown here), using resources available in the Brazilian SUS. Although the model was implemented to varying degrees at each site, it was worth pursuing based on our experience. Qualitative data captured from all sites registered that several participants were linked to a healthcare service for the first time in their lives (findings from the TransOdara study, not presented here for the sake of conciseness).

To carry out the study, it was necessary, in several sites, to include providers who initially had little experience in the care of transgender people. In turn, this required several training courses before and throughout the data collection period. In this way, our study contributed to capacity strengthening in SUS, as expanding the number of well-trained professionals to work with the transgender population is fundamental for their linkage to care, adherence, and retention to health care.

The findings of this study confirm the social vulnerability of TGW, who share intersecting stigmatized identities. The need for implementing improved public health and effective intersectoral policies that increase this population’s access to health services is clear.

## References

[1] World Health Organization (2022). Global health sector strategies on, respectively, HIV, viral hepatitis and sexually transmitted infections for the period 2022-2030.

[2] Toskin I, Blondeel K, Peeling RW, Deal C, Kiarie J (2017). Advancing point of care diagnostics for the control and prevention of STIs: the way forward. Sex Transm Infect.

[3] Unemo M, Golparian D, Eyre DW (2019). Antimicrobial resistance in neisseria gonorrhoeae and treatment of gonorrhea. Methods Mol Biol.

[4] Machado HM, Martins JM, Schörner MA, Gaspar PC, Bigolin A, Ramos MC (2022). National surveillance of *Neisseria gonorrhoeae* antimicrobial susceptibility and epidemiological data of gonorrhoea patients across Brazil, 2018-20. JAC Antimicrob Resist.

[5] Guimarães MDC, Kendall C, Magno L, Rocha GM, Knauth DR, Leal AF (2018). Comparing HIV risk-related behaviors between 2 RDS national samples of MSM in Brazil, 2009 and 2016. Medicine (Baltimore).

[6] Brasil. Ministério da Saúde (2015). Secretaria de Vigilência em Saúde. Departamento de DST, Aids e Hepatites Virais. Protocolo clínico e diretrizes terapêuticas para atenção integral às pessoas com infecções sexualmente transmissíveis.

[7] World Health Organization (2022). Consolidated guidelines on HIV, viral hepatitis and STI prevention, diagnosis, treatment and care for key populations.

[8] MacCarthy S, Poteat T, Xia Z, Roque NL, Kim AHJ, Baral S (2017). Current research gaps: a global systematic review of HIV and sexually transmissible infections among transgender populations. Sex Health.

[9] Magno L, Silva LAV, Veras MA, Pereira-Santos M, Dourado I (2019). Stigma and discrimination related to gender identity and vulnerability to HIV/AIDS among transgender women: a systematic review. Cad Saude Publica.

[10] Leite BO, Medeiros DS, Magno L, Bastos FI, Coutinho C, Brito AM (2021). Association between gender-based discrimination and medical visits and HIV testing in a large sample of transgender women in northeast Brazil. Int J Equity Health.

[11] Costa AB, Rosa HT, Pase PF, Fontanari AMV, Catelan RF, Mueller A (2018). Healthcare needs of and access barriers for brazilian transgender and gender diverse people. J Immigr Minor Health.

[12] Brasil. Ministério da Saúde. Secretaria de Vigilência em Saúde (2022). Departamento de DST, Aids e Hepatites Virais. Protocolo clínico e diretrizes terapêuticas para atenção integral às pessoas com infecções sexualmente transmissíveis.

[13] Moseholm E, Fetters MD Conceptual models to guide integration during analysis in convergent mixed methods studies. Method Innov 201.

[14] Salganik MJ (2006). Variance estimation, design effects, and sample size calculations for respondent-driven sampling. J Urban Health.

[15] Rothman KJ, Greenland S (2018). Planning study size based on precision rather than power. Epidemiology.

[16] Heckathorn DD (1997). Respondent-driven sampling: a new approach to the study of hidden populations. Social Problems.

[17] Bastos FI, Bastos LS, Coutinho C, Toledo L, Mota JC, Velasco-de-Castro CA (2018). HIV, HCV, HBV, and syphilis among transgender women from Brazil: assessing different methods to adjust infection rates of a hard-to-reach, sparse population. Medicine (Baltimore).

[18] Veras MASM, Saggese GSR, Gomez JL, Silveira P, Paiatto B, Ferreira D (2021). Brief report: young age and sex work are associated with hiv seroconversion among transgender women in São Paulo, Brazil. J Acquir Immune Defic Syndr.

[19] Gaydos CA, Cartwright CP, Colaninno P, Welsch J, Holden J, Ho SY (2010). Performance of the Abbott RealTime CT/NG for detection of Chlamydia trachomatis and Neisseria gonorrhoeae. J Clin Microbiol.

[20] Poynten IM, Jin F, Molano M, Machalek DA, Roberts JM, Hillman RJ (2022). Comparison of four assays for human papillomavirus detection in the anal canal. Clin Microbiol Infect.

[21] McCartney DJ, Pinheiro TF, Gomez JL, Carvalho PGC, Veras MA, Mayaud P (2022). Acceptability of self-sampling for etiological diagnosis of mucosal sexually transmitted infections (STIs) among transgender women in a longitudinal cohort study in São Paulo, Brazil. Braz J Infect Dis.

[22] Harris PA, Taylor R, Thielke R, Payne J, Gonzalez N, Conde JG (2009). Research electronic data capture (REDCap)--a metadata-driven methodology and workflow process for providing translational research informatics support. J Biomed Inform.

[23] R Core Team (2009). R: A language and environment for statistical computing.

[24] Avery L, Rotondi N, McKnight C, Firestone M, Smylie J, Rotondi M (2019). Unweighted regression models perform better than weighted regression techniques for respondent-driven sampling data: results from a simulation study. BMC Med Res Methodol.

[25] Sperandei S, Bastos LS, Ribeiro-Alves M, Reis A, Bastos FI (2023). Assessing logistic regression applied to respondent-driven sampling studies: a simulation study with an application to empirical data. Int J Soc Res Methodol.

[26] Christenfeld NJS, Sloan RP, Carroll D, Greenland S (2004). Risk factors, confounding, and the illusion of statistical control. Psychosom Med.

