# Supplementary material for: TransOdara study: the challenge of integrating methods, settings and procedures during the COVID-19 pandemic in Brazil
Source: Rev Bras Epidemiol. 2024 Aug 19;27(Suppl 1):e240002.supl.1. doi: 10.1590/1980-549720240002.supl.1 (PMC11338537; doi:10.1590/1980-549720240002.supl.1)
Supplement: Translation

# Translation: Estudo TransOdara: o desafio de integrar métodos, contextos e procedimentos durante a pandemia de COVID-19 no Brasil

**DOI:** 10.1590/1980-549720240002.supl.1.2

**Authors:** Maria Amelia de Sousa Mascena Veras, Thiago Felix Pinheiro, Lenice Galan, Laio Magno, Andréa Fachel Leal, Daniela Riva Knauth, Ana Rita Coimbra Motta-Castro, Rita Suely Bacuri de Queiroz, Philippe Mayaud, Daniel Jason McCartney, Gwenda Hughes, Camila Mattos dos Santos, Leonardo Bastos, Katia Cristina Bassichetto, Sandro Sperandei, Claudia Renata dos Santos Barros, Rodrigo Calado da Silva, Francisco Inácio Bastos, Maria Inês Costa Dourado

**Affiliations:** I Santa Casa de São Paulo, Escola de Ciências Médicas – São Paulo (SP), Brazil.; II Núcleo de Pesquisa e Direitos Humanos em Saúde da População LGBT+ (NUDHES) – São Paulo (SP), Brasil.; III Universidade do Estado da Bahia, Departamento de Ciências da Vida – Salvador (BA), Brasil.; IV Universidade Federal do Rio Grande do Sul – Porto Alegre (RS), Brasil.; V Universidade Federal de Mato Grosso do Sul – Campo Grande (MS), Brasil.; VI Fundação Oswaldo Cruz, Instituto Leônidas e Maria Deane – Manaus (AM), Brasil.; VI London School of Hygiene & Tropical Medicine, Faculty of Infectious & Tropical Diseases, Department of Clinical Research – London, United Kingdom.; VIII London School of Hygiene & Tropical Medicine, Faculty of Epidemiology and Public Health, Department of Infectious Diseases – London, United Kingdom.; IX Fundação Oswaldo Cruz, Programa de Computação Científica – Rio de Janeiro (RJ), Brasil.; X Western Sydney University, Translational Health Research Institute – Penrith, Australia.; XI Universidade de São Paulo, Instituto Butantan – São Paulo (SP), Brasil.; XII Santa Casa Instituto de Pesquisa – São Paulo (SP), Brasil.; XIII Fundação Oswaldo Cruz, Instituto de Comunicação e Informação Científica e Tecnológica em Saúde – Rio de Janeiro (RJ), Brasil.; XIV Universidade Federal da Bahia, Instituto de Saúde Coletiva – Salvador (BA), Brasil.

**Keywords:** Pessoas transgênero, Sífilis, HIV, Infecções sexualmente transmissíveis, Amostragem, Respondent-driven sampling, Ciência da implementação

## Abstract

**Objetivo:**

As infecções sexualmente transmissíveis (IST) afetam desproporcionalmente as *mulheres trans e travestis (MTT)*, que muitas vezes não têm acesso a cuidados de saúde devido ao estigma e à discriminação. Descrevemos a abordagem e a metodologia de um estudo que investigou a prevalência de sífilis, HIV, hepatite A, B e C, *Neisseria gonorrhoeae* (NG), *Chlamydia trachomatis* (CT) e papilomavírus humano (HPV) entre as *MTT*, bem como seu conhecimento e percepção sobre a sífilis, para melhor as políticas para redução de IST nessa população vulnerável.

**Métodos:**

TransOdara foi um estudo multicêntrico, transversal, realizado em cinco capitais das principais regiões brasileiras entre dezembro de 2019 e julho de 2021. Mulheres autoidentificadas como mulheres trans ou travestis, com idade >18 anos, foram recrutadas usando *respondent-driven sampling*, após uma fase de pesquisa formativa. Responderam a um questionário conduzido por entrevistadoras. Foi oferecida consulta médica, com exame físico, e solicitou-se que fornecessem amostras de vários locais para detectar as IST citadas. Quando indicado e consentido, foram iniciadas a vacinação e o tratamento.

**Resultados:**

Foram recrutadas 1.317 participantes nos cinco locais de estudo: Campo Grande (n=181, 13,7%), Manaus (n=340, 25,8%), Porto Alegre (n=192, 14,6%), Salvador (n= =201, 15,3%) e São Paulo (n=403, 30,6%). O período de recrutamento variou em cada local em razão de restrições logísticas impostas pela pandemia de COVID-19.

**Conclusão:**

Apesar dos enormes desafios colocados pela ocorrência simultânea da pandemia da COVID-19 e do trabalho de campo dirigido a uma população vulnerabilizada e dispersa, o projeto TransOdara foi eficazmente implementado. As adversidades não impediram que mais de 1.300 mulheres trans e travestis tenham sido entrevistadas e testadas em meio a uma epidemia de tal magnitude que perturbou os serviços de saúde e os projetos de pesquisa no Brasil e no mundo.

## INTRODUÇÃO

As Infecções Sexualmente Transmissíveis (IST) continuam a ser um grande problema de saúde pública em todo o mundo, impactando a qualidade de vida e resultando em significativa morbidade e mortalidade. De acordo com a Organização Mundial da Saúde (OMS), mais de um milhão de IST curáveis são contraídas todos os dias, causadas principalmente por *Chlamydia trachomatis* (CT), *Neisseria gonorrhoeae* (NG), *Treponema pallidum* (sífilis) e *Trichomonas vaginalis*. Além disso, um número inaceitavelmente elevado de pessoas ainda morre de HIV e hepatite B ou C^1^.

Recentemente, foram propostas estratégias para reduzir o fardo global das IST, incluindo o desenvolvimento de testes rápidos no local de atendimento, que fornecem resultados imediatamente após a coleta da amostra, evitando a perda da oportunidade de tratamento e cuidados imediatos^2^. Tais estratégias são especialmente relevantes para reduzir a incidência entre as populações que enfrentam barreiras no acesso aos cuidados e tratamento nas unidades de saúde.

Apesar desses esforços, a prevalência de IST não diminuiu, e surtos recentes de novas infecções transmitidas por contato sexual, tais como Mpox, *Shigella sonnei*, *Neisseria meningitidis*, Ébola e Zika, têm sido registrados, tornando difícil o controle dessas doenças e a sua consequências^1^. Além disso, a resistência antimicrobiana representa uma ameaça significativa à saúde pública global, particularmente para a NG, uma vez que pode levar a infecções ainda mais difíceis de tratar^3-5^.

No Brasil, os dados sobre a prevalência das IST mais comuns são limitados, provavelmente devido ao manejo sindrômico das IST implementado pelo sistema público de saúde desde o início da década de 1990. Isso resultou em notificações/vigilância etiológica limitadas de IST para fins de diagnóstico^6^.

As mulheres trans e travestis (MTT), termo abrangente que inclui todos os outros indivíduos que se identificam com uma identidade de gênero diferente do sexo masculino atribuído ao nascimento, correm um risco mais elevado de contrair HIV e outras IST. No entanto, há uma escassez de dados disponíveis sobre outras IST além do HIV para essa população, especialmente em países de baixa e média renda, como o Brasil^7,8^.

Os dados disponíveis sugerem que as taxas de IST em MTT no Brasil são altas^9^. Em um estudo realizado no Rio de Janeiro, 28,9% dos MTT foram diagnosticadas com sífilis, 14,6% com clamídia retal e 13,5% com gonorreia retal^10^, enquanto a prevalência estimada de HIV no Rio de Janeiro e em São Paulo ficou entre 30–40%^10,11^. Os determinantes da aquisição e transmissão de IST sobrepõem-se aos do HIV, incluindo relações sexuais anais receptivas sem preservativo^12^. Outros fatores de risco importantes para a infecção pelo HIV em MTT incluem abuso psicológico e físico resultante da não conformidade de gênero^13^, idade jovem, raça/cor de pele negra e histórico de trabalho sexual e/ou uso de cocaína. No entanto, são necessários dados mais robustos e oportunos sobre a frequência e os fatores de risco de IST não relacionadas com o HIV em MTT.

O estigma e a discriminação são obstáculos significativos ao acesso aos serviços de saúde para as MTT, levando a piores resultados de saúde^9,10^. Essas mulheres frequentemente relatam experiências negativas em ambientes de cuidados de saúde, tais como linguagem insensível ou recusa de cuidados, enquanto os prestadores de cuidados de saúde muitas vezes se sentem despreparados para prestar cuidados adequados^11^. Essa falta de cuidados adequados de afirmação de gênero provavelmente inibe as pessoas trans de procurarem testes, diagnóstico e tratamento para IST, bem como outros cuidados de saúde necessários.

Há uma necessidade de dados robustos sobre a epidemiologia das IST em MTT e de uma compreensão das disparidades de saúde que enfrentam. Esses dados são essenciais para informar o desenvolvimento de serviços e estratégias eficazes de prevenção e tratamento^12^.

Para abordar essas lacunas, estabelecemos o estudo TransOdara com os seguintes objetivos:

1. Estimar a prevalência de IST, incluindo sífilis, HIV, NG, CT, papilomavírus humano (HPV), vírus da hepatite A (HAV), hepatite B (HBV) e hepatite C (HCV), entre mulheres trans e travestis em cada uma das cinco macrorregiões do Brasil;
2. Demonstrar a viabilidade de implementação de tratamento e prevenção no local de atendimento no Sistema Único de Saúde (SUS); e
3. Compreender os significados atribuídos à sífilis e as vivências nos serviços de saúde entre as MTT.

Aqui apresentamos a metodologia e os procedimentos do estudo TransOdara. Descrevemos alguns dos desafios enfrentados ao estabelecer o estudo durante a pandemia de COVID-19 e como eles foram abordados.

## MÉTODOS

O TransOdara é um estudo multicêntrico e transversal realizado em cinco capitais brasileiras: Campo Grande, Manaus, Porto Alegre, Salvador e São Paulo entre dezembro de 2019 e julho de 2021, entre mulheres trans e travestis.

Este estudo de metodologia mista adotou um desenho convergente com uma estrutura bidirecional simultânea para análise de fusão de dados, que envolveu uma consideração interativa de perspectivas qualitativas e quantitativas^13^.

### Pesquisa qualitativa

O estudo começou com uma fase formativa utilizando métodos e técnicas de pesquisa complementares. Inicialmente, foram mapeados e visitados locais sociais e instituições que ofereciam serviços para pessoas trans, a fim de obter informações sobre as redes sociais das MTT e pessoas que elas conheciam. Isso ajudou na seleção das ‘sementes’ para o componente quantitativo em cada local de estudo. Em seguida, identificamos potenciais participantes, indicados por informantes-chave, e realizamos grupos focais para: compreender os significados atribuídos por mulheres trans e travestis à sífilis e a outras IST; identificar os seus conhecimentos sobre prevenção, testagem e tratamento dessas infecções; avaliar a aceitabilidade de diferentes estratégias de teste e tratamento; e compreender sua experiência com os serviços de saúde. Em São Paulo, um grupo focal com MTT foi convocado inicialmente para nomear e desenvolver a identidade visual do estudo a partir de suas referências semânticas e estéticas, de acordo com suas expectativas para um projeto de saúde (Figura 1).

**Figura 1:** Produção visual – arte produzida para comunicação, conforme projeto imagético da pesquisa.

Outro componente qualitativo foi realizado com participantes já incluídas no componente quantitativo (exceto em Salvador, onde as duas etapas qualitativas foram realizadas simultaneamente). Nessa etapa, foram realizadas entrevistas em profundidade com participantes de diferentes níveis de escolaridade, tanto aquelas que relataram diagnóstico prévio de sífilis quanto aquelas que não o fizeram. As entrevistas tiveram como objetivo compreender a vulnerabilidade das mulheres trans e travestis às IST, os significados atribuídos a tais infecções, o acesso (ou falta dele) aos serviços de saúde e os itinerários no sistema de saúde, com foco na identificação de barreiras ao tratamento das IST.

### Pesquisa quantitativa

#### Procedimentos de amostragem e tamanho da amostra

Um tamanho de amostra de 1.280 mulheres trans e travestis foi calculado *a priori* para fornecer uma estimativa da prevalência de sífilis com erros padrão para cada local, utilizando um método apropriado onde o tamanho da população é desconhecido^14^. O tamanho da amostra de cada local foi calculado para estimar a prevalência de sífilis ativa, considerando títulos >1:8 no VDRL e o tamanho das respectivas cidades, conforme observado na Tabela 1.

**Tabela 1:** Tamanho da amostra para cada local e respectivo erro padrão.

Local	Tamanho da amostra	Erro padrão (%)
Manaus	300	3,7
Salvador	200	4,7
Campo Grande	180	5,0
Porto Alegre	200	4,8
São Paulo	400	3,1

As questões operacionais e as restrições orçamentais foram considerações importantes na escolha da sífilis ativa como resultado primário para os cálculos do tamanho da amostra. Estudos anteriores sugeriram que outras IST poderiam ser menos prevalentes que a sífilis, levando a amostras muito maiores. Certamente, a nossa escolha pode resultar numa precisão inferior à ideal das estimativas de prevalência para infecções raras^15^. O impacto dessa limitação nas estimativas de ITS específicas será discutido nos documentos analíticos relevantes.

#### Recrutamento

As MTT foram recrutadas nos cinco locais de estudo através do método *respondent-driven sampling* (RDS). O RDS é uma abordagem apropriada para o recrutamento dessa população^16^ e tem sido utilizado para o recrutamento de MTT em outros estudos no Brasil^17^.

Durante a pesquisa formativa em cada local de estudo, potenciais “sementes” (sete a nove) foram selecionadas através de entrevistas em profundidade e grupos focais com membros da comunidade de mulheres trans e travestis, que foram inicialmente contatados por trabalhadoras de proximidade/pares que já trabalhavam com as equipes de pesquisa locais. Os critérios para a escolha das “sementes” incluíam ligações a uma grande rede social de potenciais participantes e garantiam diversidade suficiente em características de interesse, como o nível de escolaridade e o envolvimento no trabalho sexual.

Cada semente recebeu um número limitado de cupons (5–6) para distribuir a outras pessoas em sua rede social, e as participantes subsequentes receberam um número semelhante de cupons cada para distribuir. A metodologia RDS exige que as participantes indiquem pessoas que conhecem e com quem mantêm relacionamento social. As participantes foram instruídas a convidar outras pessoas que se identificassem como parte da mesma população de interesse do estudo. O número de seis cupons distribuídos a cada participante foi estabelecido para maximizar o recrutamento para o estudo e foi baseado na experiência anterior de pesquisa com MTT. As cadeias de recrutamento foram rastreadas usando um “gerenciador de cupons”, com cada participante recebendo um número exclusivo que identificava o local da pesquisa, o número de identificação individual e a sequência de recrutamento.

Os critérios de elegibilidade das participantes incluíam: ter 18 anos ou mais, ter sido designada no sexo masculino ao nascer, autoidentificação com identidade de gênero feminina (incluindo travesti, mulher, mulher trans, agênero ou outra identificação feminina), residir na área metropolitana dos locais do estudo e apresentar um cupom de estudo válido para participação na pesquisa.

As participantes foram reembolsadas pelas despesas com alimentação e transporte e foi necessário que fornecessem consentimento informado para participar do estudo.

Cada um dos locais de estudo teve a liberdade de adotar estratégias de recrutamento mais adequadas ao seu contexto, desde que não comprometessem os objetivos e o desenho geral do estudo. Em São Paulo, ao contrário dos outros locais, o estudo TransOdara foi integrado ao estudo de coorte TransNacional^18^, que também utilizou a metodologia RDS para recrutamento. As participantes da coorte foram convidadas a participar do estudo TransOdara e agendadas para os procedimentos propostos pelo novo estudo.

Apesar dos desafios impostos pela pandemia, toda a formação e supervisão planejadas no local foram realizadas de forma remota. As/os investigadoras/es em todos os locais de estudo possuíam experiência anterior na condução de outros estudos RDS, facilitando a transição para a formação *online* e a supervisão de todas as atividades de pesquisa.

#### Coleta de dados

Todas as participantes preencheram formulários específicos de coleta de dados incluindo:

- elegibilidade;
- aceitabilidade da coleta de amostras; e
- um questionário padrão conduzido por entrevistador/a para informações sociodemográficas com mais de duzentas perguntas relacionadas a procedimentos de afirmação de gênero, estigma e discriminação, encarceramento, saúde mental, uso de álcool e outras substâncias, comportamento sexual, acesso a cuidados de saúde e testes de HIV/IST, profilaxia pré-exposição (PrEP) e profilaxia pós-exposição (PEP) para prevenção do HIV e sintomas de IST nos últimos seis meses e no momento da visita do estudo.

As entrevistas presenciais foram realizadas por funcionárias/os bem treinadas/os, usando *laptops*.

#### Procedimentos clínicos e laboratoriais

As participantes foram solicitadas a fornecer voluntariamente amostras biológicas de vários locais do corpo para triagem de IST, com o direito de recusar qualquer um dos testes. Amostras de sangue foram coletadas e testadas para infecções por HIV, sífilis e hepatite (A, B, C), utilizando as diretrizes atuais do Ministério da Saúde do Brasil e com produtos registrados e aprovados pela Agência Nacional de Vigilância Sanitária (ANVISA). Testes rápidos para detecção de anti-HIV-1/2, anti-HCV, anti-HBsAg e anti-*Treponema pallidum* foram realizados durante a visita do estudo, seguidos de testes laboratoriais para confirmação e para determinar o tempo de infecção.

Amostras de urina, anorretal e orofaríngea foram testadas para CT e NG usando o teste de *Abbott RealTime* CT/NG (Des Plaines, IL, EUA), que já demonstrou alta precisão para cada um desses locais anatômicos para cada patógeno^19^. O *Seegene Anyplex II HPV28 Detection* (Seul, República da Coreia) foi usado para detectar o DNA do HPV e identificar genótipos específicos presentes em esfregaços da região perianal e genitais externos, com precisão demonstrada principalmente para amostras cervicais em mulheres cisgênero, mas considerada aceitável para detecção de genótipos de HPV no canal anal^20^. As participantes puderam escolher se essas amostras foram autocoletadas ou coletadas por profissional de saúde, com diagramas instrutivos desenvolvidos especificamente para o estudo, levando em consideração corpos trans, para orientar as participantes na autocoleta. Procedimentos detalhados e aceitabilidade da coleta de amostras por conta própria ou por profissional de saúde foram publicados em outro estudo^21^.

Cada participante foi solicitada a consentir a realização de um exame físico por um/a médico/a do estudo, com o objetivo de observar e relatar sinais de infecção usando um formulário padronizado de relato de caso, independentemente de quaisquer sintomas relatados. A participante poderia ainda optar por não participar de todos ou quaisquer exames. Os exames incluíam uma avaliação geral da pele, orofaringe e gânglios linfáticos axilares e inguinais (para detectar possíveis sinais de sífilis, verrugas, úlceras, inflamação e adenomegalia); exame genital (para detectar a presença de corrimento genital, verrugas e úlceras); e um exame anal (para detectar a presença de secreção anal, verrugas e úlceras). O exame genital foi baseado na genitália presente (pênis e escroto ou neovagina pós-cirurgia).

No geral, os exames clínicos e laboratoriais foram bem aceitos pelas entrevistadas e o número de recusas foi insignificante (ou seja, 98,5% consentiram a coleta de amostras biológicas).

Quando necessário, as participantes foram encaminhadas para tratamento de qualquer condição evidenciada por exames laboratoriais ou clínicos. A situação vacinal de cada participante foi avaliada e, quando indicado, foram encaminhadas para receber as vacinas nos serviços do SUS conforme indicação. Todas as participantes, independentemente da cidade, seguiram os mesmos procedimentos em relação à oferta de testes, exames e vacinas. Além disso, foram desenvolvidos procedimentos operacionais padrão (POPs) para garantir a homogeneidade entre os cinco locais de estudo. Uma gestora de dados trabalhou durante todo o período do estudo para verificar a qualidade dos dados coletados e solicitar correções ou complementações sempre que necessário.

#### Armazenamento e limpeza de dados

Todos os dados, incluindo as respostas de questionários conduzidos por entrevistadoras/es e formulários de relatos de casos, foram coletados e gerenciados como uma única entrada usando a *REDCap* ^22^, uma plataforma segura baseada na *web* projetada para apoiar a coleta de dados validados para estudos de pesquisa, hospedados na Faculdade de Ciências Médicas da Santa Casa de São Paulo. A limpeza dos dados e a análise estatística foram realizadas utilizando o R^23^. Para estimar a prevalência das IST de interesse, cada cidade foi tratada como um cluster devido ao recrutamento entre pares e à diversidade das redes. Os pesos RDS não foram utilizados na análise, pois estudos recentes demonstraram que não melhoram o desempenho do modelo e podem introduzir mais incerteza, dependendo da estrutura da rede subjacente^24,25^.

#### Aspectos éticos

O projeto foi aprovado pelo Comitê de Ética em Pesquisa (CEP) da Santa Casa de Misericórdia de São Paulo (CAAE 05585518.7.0000.5479 - Nº parecer: 3.126.815 - 30/01/2019), assim como pelas demais instituições participantes: Instituto Leônidas e Maria Deane — ILMD/Fiocruz — Manaus; Centro de Referência e Treinamento em DST/Aids, Instituto Adolfo Lutz; Secretaria Municipal de Saúde de Porto Alegre; Universidade Federal da Bahia; Universidade Federal de Mato Grosso do Sul — UFMS, Universidade Federal de Ciências da Saúde de Porto Alegre — UFCSPA e Universidade Federal do Rio Grande do Sul — UFRGS.

O consentimento livre e esclarecido por escrito foi obtido de todas as participantes individuais incluídas no estudo. Indivíduos que se identificaram com uma identidade transfeminina estiveram envolvidas na concepção e implementação do estudo e lideraram o recrutamento de participantes utilizando RDS.

## RESULTADOS

Foram recrutadas 1.317 participantes nos cinco locais de estudo: Campo Grande (n=180, 13,7%), Manaus (n=333, 25,8%), Porto Alegre (n=191, 14,6%), Salvador (n=207, 15,3%) e São Paulo (n=406, 30,6%). O período de recrutamento variou em cada local de estudo, devido aos diferentes prazos para obtenção da aprovação ética e às consequências das ondas epidemiológicas da pandemia de COVID-19. Em diferentes fases da curva epidemiológica, medidas como aconselhar as pessoas a ficarem em casa, restringir o acesso a unidades de saúde primárias (em quatro de cada cinco locais) apenas a emergências, e fechar *campi* universitários, entre outras medidas, afetaram diretamente as atividades de pesquisa.

A idade das participantes variou de 18 a 68 anos, com média de idade de 30 anos (desvio padrão — DP=9,9), variando conforme as cidades: 29 — DP=10,7 (Campo Grande), 30 — DP 9,3 (Manaus), 32 — DP=9,9 (Porto Alegre), 28 — DP=9,6 (Salvador) e 33 — DP=9,7 (São Paulo). Idade mediana de 31 anos (intervalo interquartil — AIQ 24–38). A maioria das participantes identificou-se como tendo raça/cor de pele ‘parda’ (44%), com proporções semelhantes relatando ‘preta’ ou ‘branca’ (27 e 26%, respectivamente). A maioria das participantes se identificou como mulher transexual (56,3%) ou travesti (29,8%).

Menos de um terço (29,3%) das participantes relatou ter mudado o nome em algum documento oficial. Mais de um terço das participantes relataram não ter religião (36%). O catolicismo foi a religião mais citada (26%), seguida pelas afro-brasileiras (22%). A maioria referiu ter ensino secundário (58%), enquanto um quarto (25%) reportou ter apenas o nível primário ou nenhuma escolaridade.

Uma percentagem substancial de participantes (37,6%) vivia em condições instáveis (como abrigos ou dormindo no local de trabalho), incluindo pessoas sem-teto (1,8%). A situação profissional variou, com a maior proporção sendo de desempregadas (22,3%), seguida de trabalhadoras do sexo (21,3%) ou trabalhadoras independentes (16,1%). Apenas uma pequena proporção relatou estar formalmente empregada (8,4%). A renda mensal da maioria das participantes (57%) era de até um salário mínimo — em reais (BRL), aproximadamente 1.000,00 (R$ 998,00 em 2019, 1.100,00 em 2021, ou aproximadamente US$ 200).

Mais de um quarto (27%) das participantes relatou ter sido submetida a algum procedimento ou cirurgia relacionado à transição, enquanto uma proporção muito pequena (1,7%) relatou ter uma neovagina após ser submetida a uma cirurgia para remover o pênis e o escroto. A maioria das participantes (86%) já usou hormônios antes, enquanto pouco menos da metade (48%) estava usando hormônios para afirmação de gênero.

O estudo constatou altos níveis de discriminação e violência, com a maioria das participantes relatando ter sofrido algum tipo de discriminação (85,0%) ou sexo forçado (51%). Quase um quarto (23%) relatou ter sido presa e, entre essas, menos de 1% (0,8%) foi colocada em uma cela dedicada a pessoas LGBTQI+.

A maioria das participantes relatou sua orientação sexual como heterossexual (78,7%) e seu *status* de parceria como solteira (69,9%). Nos últimos seis meses, menos de metade relatou ter um parceiro sexual fixo (48,6%) ou parceiros sexuais casuais (44,0%). Enquanto dois quintos (39,7%) indicaram ter tido pelo menos um parceiro sexual comercial nos últimos seis meses, um quinto (21,3%) relatou o trabalho sexual como a sua principal fonte de renda. A maioria das participantes relatou ter praticado sexo transacional (73,7%).

As características das participantes por local e a amostra total são apresentadas na Tabela 2.

**Tabela 2:** Características sociodemográficas das mulheres trans e travestis participantes do estudo TransOdara, segundo cidades de estudo. Dezembro de 2019 a julho de 2021.

Variáveis/locais	Locais de pesquisa e Brasil					
	Campo Grande (n=180)	Manaus (n=333)	Porto Alegre (n=191)	Salvador (n=207)	São Paulo (n=406)	Total (n=1.317)
	n (%)	n (%)	n (%)	n (%)	n (%)	n (%)
Dados sociodemográficos						
Identidade de gênero						
Mulher	12 (6,8)	4 (1,2)	21 (11)	22 (11)	39 (9,6)	98 (7,4)
Mulher transexual	78 (43)	172 (52)	96 (50)	130 (63)	265 (65)	741 (56)
Travesti	59 (32,8)	150 (45)	48 (25)	40 (19)	96 (24)	393 (30)
Transexual	9 (5,0)	0 (0)	18 (9,4)	7 (3,4)	4 (1,0)	38 (2,9)
Outro	22 (12,4)	7 (2,1)	8 (4,2)	8 (3,9)	2 (0,5)	47 (3,7)
Tentativa de mudança de nome						
Não sentiu necessidade	33 (24)	208 (68)	20 (19)	46 (30)	46 (20)	353 (38)
Não sabia ter direito	1 (0,7)	30 (9,8)	3 (2,8)	6 (3,9)	4 (1,7)	44 (4,7)
Não sabia como fazê-lo	6 (4,4)	29 (9,5)	16 (15)	13 (8,6)	13 (5,7)	77 (8,3)
Achou ser muito caro	12 (8,8)	8 (2,6)	9 (8,3)	6 (3,9)	41 (18)	76 (8,2)
Achou ser muito complicado	11 (8,1)	11 (3,6)	13 (12)	35 (23)	50 (22)	120 (13)
Outros motivos/não respondeu	73 (54)	19 (6,2)	47 (44)	46 (30)	76 (33)	261 (28)
Raça/cor da pele						
Branca	55 (32)	60 (18)	91 (48)	22 (11)	108 (27)	336 (26)
Preta	38 (22)	52 (16)	60 (31)	107 (52)	92 (23)	349 (27)
Amarela	2 (1,2)	12 (3,6)	3 (1,6)	4 (1,9)	5 (1,2)	26 (2,0)
Parda	1 (0,6)	8 (2,4)	1 (0,5)	2 (1,0)	7 (1,7)	19 (1,5)
Indígena	76 (44)	199 (60)	36 (19)	72 (35)	192 (47)	575 (44)
Religião						
Sem religião	70 (40)	100 (30)	62 (33)	92 (44)	151 (37)	475 (36)
Afro-Brasileiro	24 (14)	37 (11)	83 (44)	73 (35)	69 (17)	286 (22)
Evangélica	18 (10)	33 (9,9)	7 (3,7)	6 (2,9)	52 (13)	116 (8,8)
Católica	49 (28)	149 (45)	26 (14)	31 (15)	91 (22)	346 (26)
Espírita	14 (8,0)	12 (3,6)	10 (5,3)	4 (1,9)	35 (8,6)	75 (5,7)
Judia	0 (0)	0 (0)	0 (0)	0 (0)	2 (0,5)	2 (0,2)
Oriental	1 (0,6)	1 (0,3)	2 (1,1)	1 (0,5)	4 (1,0)	9 (0,7)
Nível educacional						
Até o ensino fundamental	48 (27)	76 (23)	52 (27)	55 (27)	97 (24)	328 (25)
Até o ensino médio e curso técnico	95 (53)	201 (61)	97 (51)	117 (57)	253 (62)	763 (58)
Ensino superior e pós-graduação	35 (20)	55 (17)	42 (22)	34 (16)	56 (14)	222 (17)
Moradia						
Mora em casa/apartamento próprio	44 (25)	50 (15)	63 (33)	71 (34)	115 (28)	343 (26)
Mora em casa ou apartamento alugado	60 (34)	82 (25)	65 (34)	94 (45)	179 (44)	480 (36)
Mora provisoriamente com família/amigo/na rua	61 (34)	171 (51)	52 (27)	31 (15)	52 (13)	367 (28)
Orientação sexual						
Heterossexual	106 (59)	288 (87)	127 (66)	168 (81)	347 (85)	1.036 (79)
Homossexual, *gay*, ou lésbica	34 (19)	23 (6,9)	17 (8,9)	7 (3,4)	16 (3,9)	97 (7,4)
Bissexual	20 (11)	15 (4,5)	17 (8,9)	10 (4,8)	23 (5,7)	85 (6,5)
Pansexual	15 (8,4)	5 (1,5)	26 (14)	19 (9,2)	16 (3,9)	81 (6,2)
Assexual	1 (0,6)	0 (0)	1 (0,5)	0 (0)	1 (0,2)	3 (0,2)
Ocupação principal						
Atualmente não trabalha/desempregada	27 (15)	113 (34)	49 (26)	36 (17)	69 (17)	294 (22)
Empregada com carteira assinada	9 (5,0)	15 (4,5)	16 (8,4)	10 (4,8)	61 (15)	111 (8,4)
Empregada sem carteira assinada	23 (13)	56 (17)	10 (5,2)	21 (10)	40 (9,9)	150 (11)
Autônoma	19 (11)	39 (12)	45 (24)	55 (27)	54 (13)	212 (16)
Trabalhos esporádicos/bicos	5 (2,8)	37 (11)	7 (3,7)	22 (11)	39 (9,6)	110 (8,4)
Profissional do sexo	54 (30)	42 (13)	38 (20)	57 (28)	89 (22)	280 (21)
Outro	42 (23)	30 (9,0)	26 (14)	6 (2,9)	54 (13)	158 (12)
Renda mensal						
Média	1.939,8	1.228	1.264,6	1.564,8	1.569,8	1.499,3
Desvio padrão	1.840,3	2.772,8	1.306,1	1.480,5	3.943,1	2.810,8
Renda em salários-mínimos brasileiros						
Até 1	61 (36)	183 (69)	97 (53)	125 (66)	216 (55)	682 (57)
1–2	66 (39)	61 (23)	50 (27)	41 (22)	129 (33)	347 (29)
2–3	18 (11)	9 (3,4)	21 (12)	11 (5,8)	31 (7,9)	90 (7,5)
4+	24 (14)	11 (4,2)	14 (7,7)	12 (6,3)	17 (4,3)	78 (6,5)
Mudanças corporais						
Usou hormônios						
Não	37 (21)	78 (23)	31 (16)	17 (8,2)	20 (4,9)	183 (14)
Sim	142 (79)	253 (76)	160 (84)	190 (92)	386 (95)	1.131 (86)
Usa hormônios atualmente						
Não	87 (61)	159 (63)	82 (51)	77 (41)	186 (48)	591 (52)
Sim	55 (39)	93 (37)	77 (48)	113 (59)	198 (52)	536 (48)
Possui silicone industrial injetado						
Não	117 (65)	293 (89)	141 (74)	152 (73)	248 (61)	951 (72)
Sim	62 (35)	35 (11)	50 (26)	53 (26)	158 (39)	358 (27)
Fez alguma cirurgia para mudança corporal						
Não	139 (78)	315 (95)	97 (51)	165 (80)	234 (58)	950 (72)
Sim	40 (22)	14 (4,2)	93 (49)	40 (19)	172 (42)	359 (27)
Fez cirurgia genital						
Não	179 (100)	327 (98)	186 (97)	205 (99)	393 (97)	1.290 (98)
Sim	0 (0)	2 (0,6)	5 (2,6)	2 (1,0)	13 (3,2)	22 (1,7)
Foi discriminada						
Não	33 (18)	50 (15)	23 (12)	36 (17)	48 (12)	190 (14)
Sim	146 (82)	281 (84)	168 (88)	171 (83)	354 (88)	1.120 (85)
Sofreu alguma agressão						
Não	91 (51)	197 (60)	82 (43)	110 (53)	202 (50)	682 (52)
Sim	87 (49)	128 (39)	109 (57)	96 (46)	202 (50)	622 (48)
Foi forçada a fazer sexo						
Não	97 (54)	175 (53)	87 (46)	90 (43)	193 (48)	642 (49)
Sim	82 (46)	153 (47)	103 (54)	116 (56)	213 (52)	667 (51)
Comportamento sexual						
Companheiro estável nos últimos 6 meses						
Não	90 (51)	229 (69)	83 (43)	86 (42)	184 (45)	672 (51)
Sim	88 (49)	102 (31)	108 (57)	120 (58)	222 (55)	640 (49)
Parceiro casual nos últimos 6 meses						
Não	93 (52)	225 (68)	76 (40)	101 (49)	233 (58)	728 (55,6)
Sim	86 (48)	104 (31)	114 (60)	106 (51)	169 (42)	579 (44)
Parceiro comercial nos últimos 6 meses						
Não	89 (50)	255 (77)	88 (46)	107 (52)	246 (61)	785 (60)
Sim	90 (50)	75 (23)	101 (53)	99 (48)	158 (39)	523 (40)

## DISCUSSÃO

O estudo TransOdara foi o primeiro grande estudo no Brasil projetado para mulheres trans e travestis testando oito diferentes IST, incluindo HIV, sífilis, hepatite A, hepatite B, hepatite C, HPV, gonorreia e clamídia. As características das participantes confirmam que um número substancial de MTT enfrenta vulnerabilidade social, discriminação e barreiras no acesso aos seus direitos.

O estudo concluiu que a autoamostragem para diagnóstico etiológico de IST em locais potenciais de infecção era altamente aceitável entre mulheres trans e travestis, e poderia ser uma ferramenta importante na prevenção e tratamento dessas infecções nessa população. A aceitabilidade foi facilitada por uma equipe de profissionais amigável e bem treinada^21^.

Apesar das interrupções e atrasos causados pela pandemia da COVID-19, o estudo foi realizado com sucesso. Em São Paulo, único local que já havia sido iniciado (3 de dezembro de 2019) quando a pandemia surgiu no Brasil, o estudo atingiu metade da amostra alvo quando o trabalho de campo foi interrompido em 18 de março de 2020. O estudo foi reiniciado quatro meses depois, mas não foi possível manter a cadeia original de recrutamento do RDS e houve limitações no uso da análise de rede. O estudo foi retomado sob um protocolo rígido, incluindo contato prévio por telefone para avaliação de suspeita de infecção por SARS-CoV-2 entre as participantes. A coleta de dados durou até 29 de outubro de 2020.

Embora o RDS possa recrutar eficientemente um grande número de participantes num período relativamente curto e a um custo baixo, também pode resultar em viés de amostragem e seleção. Como o processo de recrutamento depende de redes sociais, a amostra pode não ser totalmente representativa e pode excluir subgrupos específicos. Além disso, as participantes que optaram por participar podem ter características específicas diferentes daquelas que não o fizeram. Neste estudo, por exemplo, dependendo das “sementes” selecionadas para recrutamento em cada local, as pessoas envolvidas no trabalho sexual podem estar super-representadas em alguns ou em todos os locais. Além disso, as diferenças nas características da população em vários locais podem dificultar a comparação dos resultados, sendo necessária uma interpretação cuidadosa, especialmente ao relatar a prevalência global. A mitigação desses vieses pode ser alcançada através de várias abordagens estatísticas para levar em conta os efeitos do RDS^24^.

Até onde sabemos, este é o primeiro estudo multicêntrico direcionado a uma população trans e travesti vulnerabilizada e dispersa em meio à pandemia de COVID-19. Estamos cientes sobre as limitações impostas pela pandemia e os prejuízos que as interrupções nas cadeias de recrutamento fraturadas podem causar. Conforme discutido por Christenfeld et al.^26^, a ciência não deve ser vista como uma fonte inesgotável de “truques de mágica”. No entanto, as cadeias de referência fragmentadas nunca serão tão eficazes quanto redes originais, e uma epidemia mortal deve ser abordada em primeiro lugar, como foi o caso deste estudo.

As participantes deram feedback positivo significativo sobre os serviços recebidos da estratégia no local de atendimento que integra testagem, tratamento e prevenção, incluindo início da PrEP e vacinas (dados não mostrados aqui), utilizando recursos disponíveis no SUS. Embora o modelo tenha sido implementado em graus variados em cada local, valeu a pena segui-lo com base em nossa experiência. Os dados qualitativos recolhidos em todos os locais registaram que várias participantes estiveram ligadas a um serviço de saúde pela primeira vez nas suas vidas (resultados do estudo TransOdara, não apresentados aqui por uma questão de concisão).

Para a realização do estudo foi necessário, em vários locais, incluir profissionais que inicialmente tinham pouca experiência no atendimento de pessoas trans. Por sua vez, isso exigiu diversos cursos de formação antes e durante o período de coleta de dados. Dessa forma, nosso estudo contribuiu para o fortalecimento de capacidades no SUS, uma vez que ampliar o número de profissionais bem capacitados/as para atuar com a população trans é fundamental para sua vinculação ao cuidado, adesão e retenção aos cuidados de saúde.

As conclusões deste estudo confirmam a vulnerabilidade social das MTT, que partilham identidades estigmatizadas que se cruzam. É evidente a necessidade de implementar uma saúde pública melhorada e políticas intersetoriais eficazes que aumentem o acesso dessa população aos serviços de saúde.
