# Supplementary material for: Erratum
Source: Rev Bras Epidemiol. 2024 Dec 9;27:e2023478erratum. doi: 10.1590/1980-549720240002.supl.1erratum (PMC11656493; doi:10.1590/1980-549720240002.supl.1erratum)
Supplement: Related texts

# Correction: Errata

**DOI:** 10.1590/1980-549720240002.supl.1.2erratum

No artigo "*Estudo TransOdara: o desafio de integrar métodos, contextos e procedimentos durante a pandemia de COVID-19 no Brasil*", DOI: https://doi.org/10.1590/1980-549720240002.supl.1.2, publicado no periódico Rev Bras Epidemiol. 2024; 27(Suppl 1): e240003.supl.1

**Onde se lia:**

Maria Inês Costa Dourado

**Leia-se:**

Inês Dourado
